# Thiazole Derivatives as Modulators of GluA2 AMPA Receptors: Potent Allosteric Effects and Neuroprotective Potential

**DOI:** 10.3390/biom13121694

**Published:** 2023-11-23

**Authors:** Mohammed Hawash

**Affiliations:** Department of Pharmacy, Faculty of Medicine and Health Sciences, An-Najah National University, Nablus P.O. Box 7, Palestine; mohawash@najah.edu; Tel.: +970-256-993-9939

**Keywords:** thiazole, AMPA, GluA2, neurological disorders, cytotoxicity

## Abstract

Thiazole carboxamide derivatives were synthesized in this investigation, with a subsequent examination of their impact on GluA2 AMPA receptors. The synthesized compounds, namely MMH-1-5, were subjected to characterization using high-resolution mass spectrometry (HRMS), proton nuclear magnetic resonance (^1^H-NMR), and carbon-13 nuclear magnetic resonance (^13^C-NMR). The present work thoroughly investigates the impact of five thiazole derivatives on GluA2 AMPA receptors. This investigation examined their effects on both whole-cell currents and receptor kinetics. In addition, the cytotoxicity of the samples was assessed using the MTS test. The compound **MMH-5** had the highest effect level, resulting in a notable drop in current amplitude by a factor of six. Similarly, **MMH-4** and **MMH-3** also caused major reductions in the current amplitude. The compounds mentioned above also influenced the rates of deactivation and desensitization. **MMH-5** and **MMH-4** exhibited an increase in deactivation, while **MMH-5** showed reduced desensitization. Our research findings highlight the efficacy of **MMH-5** as a negative allosteric modulator of GluA2 AMPA receptors, exerting substantial effects on both the magnitude and time course of receptor activity. Significantly, the compound MMH-2 demonstrated noteworthy cytotoxic effects, as evidenced by cell viability rates dropping below 6.79% for all cancer cell lines and 17.52% for the normal cell line (LX-2). Of particular interest is the pronounced cytotoxicity observed in **MMH-5**, suggesting its potential as a safe neuroprotective agent targeting the AMPA receptor, as indicated by cell viability percentages exceeding 85.44% across all cancer and normal cell lines. Docking simulations were performed to determine possible modes of interaction between MMH5 and the GluA2-AMPA receptor (PDB:7RZ5). The abovementioned facts and the well-documented effects of further thiazole derivatives provide a strong foundation for future research endeavors to enhance tailored treatments for neurological disorders that rely heavily on GluA2 signaling. The present study elucidates the intricate association between thiazole derivatives and GluA2 receptors, providing valuable perspectives on the prospects of enhanced and specific therapeutic interventions for diverse neurological conditions.

## 1. Introduction

Ionotropic glutamate receptors are the linchpins of fast excitatory synaptic transmission in the central nervous system (CNS). Among the subclasses of these receptors, the α-amino-3-hydroxy-5-methyl-4-isoxazolepropionic acid (AMPA) receptors hold a particularly critical role [1,2]. These tetrameric proteins are complex assemblies, with each receptor comprising a combination of four subunits—GluA1 to GluA4 [3]. In the typical hippocampus, the predominant configuration of synaptic AMPA receptors consists of heteromeric complexes formed by GluA1 and GluA2 subunits, with a significantly smaller proportion containing GluA2 and GluA3 subunits [4]. Each subunit contributes specialized characteristics to the receptor’s overall function, which include ion selectivity, conductance properties, and drug sensitivities [5].

The GluA2 subunit distinguishes itself in several key aspects: it modifies the AMPA receptor’s ion channel to be impermeable to calcium, thereby shaping synaptic strength and influencing long-term potentiation and depression. Positive allosteric modulators can enhance GluA2 currents through two distinct mechanisms. For instance, substances like aniracetam primarily work to stabilize the closed clamshell conformation when agonists are bound, preventing deactivation. On the other hand, modulators like cyclothiazide mainly function by stabilizing the receptor, thereby slowing down the process of desensitization [6,7]. By not engaging in direct competition with endogenous neurotransmitters, the negative allosteric regulation of the AMPA receptor enables precise control over the frequency and amplitude of excitatory neural activity. This control remains unaffected by variations in neurotransmitter concentrations or the polarization state of the synaptic membrane [8]. The application of negative allosteric modulation on AMPA receptors proves beneficial in managing neurological disorders associated with excessive glutamatergic activity. This approach involves the use of compounds such as 2,3-benzodiazepines, quinazolinones, and pyridines [9,10,11]. Notably, benzodiazepines are frequently employed in the management of psychiatric disorders, making them a widely recognized option in this context [12].

Furthermore, the GluA2 subunit is critical in receptor trafficking, which has downstream effects on synaptic plasticity and network function [13,14]. The physiological and pathological significance of the GluA2 subunit is vast. Dysfunctions or alterations in GluA2 expression have been linked to various neurological conditions, including but not limited to Alzheimer’s disease, epilepsy, and ischemic stroke [15,16]. Additionally, the unique kinetic properties of AMPA receptors that contain GluA2 subunits warrant particular attention. Specifically, these subunits influence the receptor’s desensitization and deactivation kinetics. Desensitization is the process through which receptors lose sensitivity due to continuous agonist binding, while deactivation is characterized by the cessation of ionic current upon the removal of the agonist [17,18,19]. The dynamics of these processes can markedly influence the strength and duration of synaptic signaling. A thorough comprehension of the kinetic properties of GluA2-containing AMPA receptors is vital for understanding the intricacies of synaptic behavior and neural networks and is crucial for the rational design of therapeutic interventions aimed at modulating these receptors [20].

**Figure 1:**
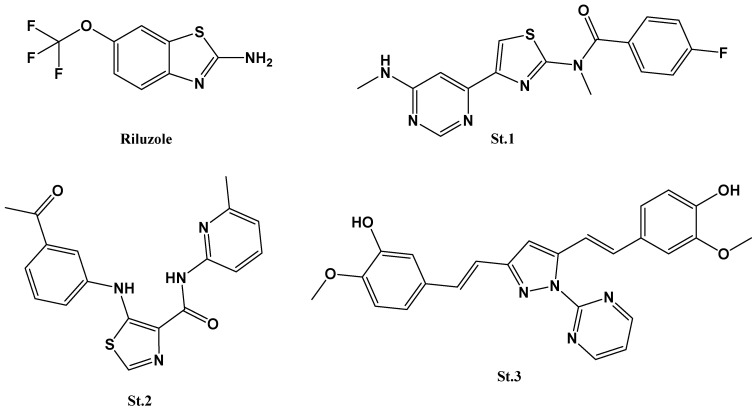
Structures of thiazole and methoxyphenyl containing compounds with protentional AMPA receptor activities.

Building on the physiological and pathological significance of GluA2-containing AMPA receptors, exploring compounds that can modulate their behavior is essential. Our study focuses on novel thiazole carboxamide derivatives; these compounds bear a close relationship to existing pharmacologically active agents as they share the thiazole ring found in thiazole derivatives such as vitamin B1 (thiamine); dasatinib, an anticancer drug [21]; and Riluzole (Figure 1), known for its neuroprotective effects in conditions like amyotrophic lateral sclerosis [22,23]. In a separate study, a series of thiazolamide derivatives were synthesized and assessed for their potential as mGluR1 antagonists. Notably, **St.1** (Figure 1) exhibited strong activity against mGluR1, yet faced challenges related to inadequate metabolic stability and limited water solubility [24]. Buettelmann and colleagues revealed a collection of thiazole-4-carboxamide derivatives in a patent application, positioning them as mGluR5a receptor antagonists. These derivatives underwent various substitutions on the thiazole carboxamide core to identify potent mGluR5a receptor antagonists. Among the compounds in this series, **St.2** (Figure 1) emerged as the most active, demonstrating a favorable mGluR5a binding affinity with a Ki value of 18 nM [25]. Previously, our research team synthesized a heterocyclic-based curcumin compound featuring methoxy substitutions on the phenyl ring. These compounds exhibited substantial activity on GluA2 receptor subtypes, notably exemplified by **St.3** (Figure 1) [15].

Importantly, some structurally similar thiazole derivatives, including Riluzole, have already been studied for their interactions with AMPA receptors [25,26]. Beyond their influence on AMPA receptors, these compounds exhibit additional pharmacological properties such as anti-inflammatory, anticancer, and neuroprotective effects [27,28]. This multidimensional therapeutic portfolio hints at a broad potential application in neurological disorders. However, the direction in which we steer the spotlight is toward understanding the kinetic subtleties of GluA2-containing AMPA receptors—focusing on how agonists and antagonists influence their molecular performance. These findings pave the way for comprehensive efforts regarding the development of negative allosteric modulators and anchoring the explorations in the structure–activity relationship (SAR), to dissect the precise mechanisms by which thiazole derivatives alter AMPA receptor behavior and in silico techniques to investigate possible binding interactions between the most potent compound and the AMPA-GluA2 target accordingly. Moreover, the cytotoxic activities of the thiazole derivatives were evaluated on various cell lines. This exploration aimed to identify more effective therapies for neurological conditions in which GluA2 signaling plays a pivotal role.

## 2. Materials and Methods

### 2.1. Materials

The chemicals used in this study were obtained from well-regarded suppliers, such as Chemicals Company, Sigma-Aldrich, and Alfa Aesar. The following substances were used in their original state, eliminating the need for additional purification: 2-(3,4-dimethoxyphenyl)-4-methylthiazole-5-carboxylic acid (Sigma-Aldrich, Burlington, MA, USA; catalog # BOG00154); 1-(3-dimethylaminopropyl)-3-ethylcarbodiimide hydrochloride (EDC) (Alfa Aesar; Karlsruhe, Germany; catalog # A10807); 4-(dimethylamino)pyridine (DMAP) (Sigma-Aldrich; Gillingham, UK; catalog # 39405-50G); 3,4,5-trimethoxyaniline (Sigma Aldrich, Burlington, MA, USA; catalog # T68209-10G), silica gel (Sigma-Aldrich; Shanghai, China; catalog # S74874); 4-tert-butylaniline (Sigma Aldrich; Germany; catalog # 209864); 3,4-dimethoxyaniline (Sigma-Aldrich; Taufkirchen, Germany; catalog # A83008); 3,5-dimethoxyaniline (Sigma-Aldrich; Taufkirchen, Germany; catalog # D130001); and 4-methylthioaniline (Sigma-Aldrich; Burlington, MA, USA; catalog # L04950). The experimental requirements were DMEM media (Sigma-Aldrich; Burlington, MA, USA; catalog # D0822), L-glutamine solution (Sigma-Aldrich; Burlington, MA, USA; catalog #D0822), and dinitrosalicylic acid (DNSA) (Sigma-Aldrich; Burlington, MA, USA; catalog #128848).

### 2.2. Instrumentation

The NMR spectra of the synthesized thiazole derivatives were scrutinized using a Bruker DPX-500 High-Performance Digital FT-NMR Spectrometer (Billerica, MA, USA) in DMSO-d_6_ solutions at the Faculty of Science, University of Jordan, Jordan. The ^1^H-NMR spectra were obtained at a 500 MHz frequency, and the ^13^C-NMR spectra were acquired at 125 MHz. Chemical shift values (δ) were measured in parts per million (ppm), with coupling constants specified in Hertz (Hz). High-resolution mass spectra (HRMS) data were generated using a water LCT Premier XE Mass Spectrometer (Waters Corporation, Milford, MA, USA) employing the positive polarity mode’s electrospray ionization (ESI) technique. This research occurred at the Pharmacy Faculty of Gazi University in Ankara, Turkey.

### 2.3. General Procedure for the Synthesis of Thiazole Carboxamide (MMH1-5)

**Scheme 1:**
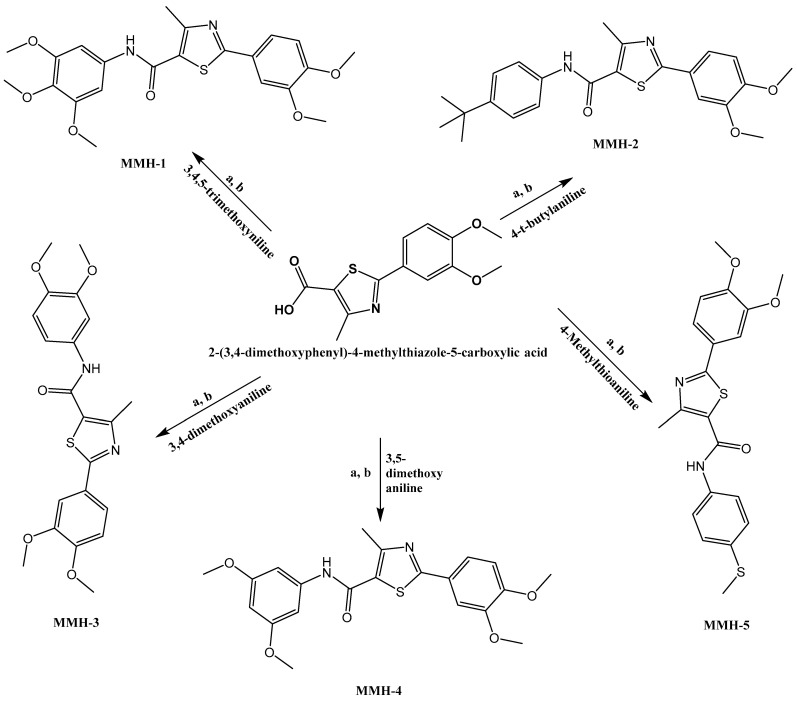
Upon stirring 2-(3,4-dimethoxyphenyl)-4-methylthiazole-5-carboxylic acid starting materials along with each aniline derivative within a 15 mL volume of DCM, the subsequent steps were executed as follows: (a) the introduction of DMAP and (b) the addition of EDC. Both steps were carried out under an inert gas environment.

Following Scheme 1, in a clean, round-bottom flask, 2-(3,4-dimethoxyphenyl)-4-methylthiazole-5-carboxylic acid (300 mg, 1.074 mmol) was dissolved in 15 mL of dichloromethane (DCM). Subsequently, dimethylaminopyridine (DMAP) (45 mg, 0.361 mmol) was introduced, and the mixture was stirred under an argon atmosphere to prevent oxidation. After 5 to 10 min, a coupling reagent, 1-ethyl-3-(3-dimethylaminopropyl)carbodiimide (EDCI, 305.91 mg, 1.5639 mmol), was added and stirred under argon. Then, following 30 min, the aniline derivative was incorporated, and the reaction blend was stirred with a mechanical stirrer for 48 h. Thin-layer chromatography (TLC) papers were treated with ninhydrin to identify the presence of aniline, while another set of papers was treated with bromocresol to confirm the presence of acid, ensuring the purity of the product. Subsequently, the reaction mixture was subjected to an extraction process with diluted hydrochloric acid in a separatory funnel. The lower layer was collected in a conical flask. Anhydrous sodium sulfate was introduced as a drying agent, and the mixture was filtered through filter paper. Silica gel (approximately 3–4 spatulas) was then added to the filtrate, and the rotary vacuum evaporator was employed to remove the reaction mixture, taking into account the boiling point of DCM at 39.6 °C. This process resulted in a flask containing silica loaded with the product. The product-loaded silica was further purified through silica gel column chromatography using a solvent system of DCM and ethyl acetate in a 2:1 ratio. The elution was collected in tubes, and the solvent evaporated [29,30].

2-(3,4-Dimethoxyphenyl)-4-methyl-N-(3,4,5-trimethoxyphenyl)thiazole-5-carboxamide (MMH1)

For the compound 2-(3,4-dimethoxyphenyl)-4-methyl-N-(3,4,5-trimethoxyphenyl)thiazole-5-carboxamide (MMH1), its ^1^H NMR (500 MHz, DMSO) is δ 10.08 (s, 1H, N**H**-amide), 7.54 (d, *J* = 8 Hz, 1H, Ar-**H**), 7.50 (s, 1H, Ar-**H**), 7.14 (s, 2H, Ar-**H**), 7.10 (d, *J* = 8.5 Hz, 1H, Ar-**H**), 3.87, 3.85 (s, 6H, -O-C**H_3_**), 3.77 (s, 6H, -O-C**H_3_**), 3.65 (s, 3H, -O-C**H_3_**), and 2.65 (s, 3H, thiazole-C**H_3_**), and the ^13^C NMR (DMSO) is δ 166.80 (C=O), 160.18, 156.44, 153.10, 151.74, 149.55, 135.29, 134.38, 125.62, 125.29, 120.13, 112.48, 109.34, 98.54, 60.58 (-O-CH_3_), 56.22 (-O-**C**H_3_), 56.17 (-O-**C**H_3_), 56.07 (-O-**C**H_3_), and 17.68 (thiazole-**C**H**_3_**). HRMS (*m*/*z*): [M + H]+ is calculated for C_22_H_24_N_2_O_6_S as 445.1430, which is found to be 445.1436.

N-(4-(*Tert*-butyl)phenyl)-2-(3,4-dimethoxyphenyl)-4-methylthiazole-5-carboxamide (MMH2)

For the compound N-(4-(tert-butyl)phenyl)-2-(3,4-dimethoxyphenyl)-4-methylthiazole-5-carboxamide (MMH2), its ^1^H NMR (500 MHz, DMSO) is δ 10.12 (s, 1H, N**H**-amide), 7.61 (d, *J* = 10.5 Hz, 2H, Ar-**H**), 7.55 (d, *J* = 10.5 Hz, 1H, Ar-**H**), 7.50 (s, 1H, Ar-**H**), 7.38 (d, *J* = 10.5 Hz, 2H, Ar-**H**), 7.11 (s, 1H, Ar-**H**), 3.87 (s, 3H, -O-C**H_3_**), 3.85 (s, 3H, -O-C**H_3_**), 2.64 (s, 3H, thiazole-C**H_3_**), and 1.29 (s, 9H, *t*-butyl), and the ^13^C NMR (DMSO) is δ 166.80 (C=O), 160.22, 160.21, 155.98, 155.97, 151.73, 149.57, 146.84, 136.56, 125.77, 120.64, 120.15, 112.52, 109.40, 56.18 (-O-**C**H_3_), 56.09 (-O-**C**H_3_), 34.55 (**C**-(CH_3_)_3_), 31.65 (C-(**C**H_3_)_3_), and 17.60 (thiazole-**C**H**_3_**). HRMS (*m*/*z*): [M + H]+ is calculated for C_23_H_26_N_2_O_3_S as 411.1750, which is found to be 411.1743.

*N*,2-bis(3,4-dimethoxyphenyl)-4-methylthiazole-5-carboxamide (MMH3)

For the compound N,2-bis(3,4-dimethoxyphenyl)-4-methylthiazole-5-carboxamide (MMH3), its ^1^H NMR (500 MHz, DMSO) is δ 10.03 (s, 1H, N**H**-amide), 7.55 (dd, *J* = 10.5, 2 Hz, 1H, Ar-**H**), 7.50 (d, *J* = 2.5 Hz, 1H, Ar-**H**), 7.39 (d, *J* = 3 Hz, 1H, Ar-**H**), 7.25 (dd, *J* = 8, 3 Hz, 1H, Ar-**H**), 7.11 (d, *J* = 10.5 Hz, 1H, Ar-**H**), 6.94 (d, *J* = 10.5 Hz, 1H, Ar-**H**), 3.87, 3.85 (s, 6H, -O-C**H_3_**), 3.75 (s, 6H, -O-C**H_3_**), and 2.65 (s, 3H, thiazole-C**H_3_**), and the ^13^C NMR (DMSO) is δ166.67 (C=O), 160.00, 156.06, 151.70, 149.55, 148.92, 145.86, 132.64, 125.67, 125.55, 120.11, 112.88, 112.49, 112.34, 109.33, 105.97, 56.18 (-O-**C**H_3_), 56.07 (-O-**C**H_3_), 55.88 (-O-**C**H_3_), and 17.63 (thiazole-**C**H**_3_**). HRMS (*m*/*z*): [M + H]+ is calculated for C_21_H_22_N_2_O_5_S as 415.1340, which is found to be 415.1348.

2-(3,4-Dimethoxyphenyl)-N-(3,5-dimethoxyphenyl)-4-methylthiazole-5-carboxamide (MMH4)

For the compound 2-(3,4-dimethoxyphenyl)-N-(3,5-dimethoxyphenyl)-4-methylthiazole-5-carboxamide (MMH4), its ^1^H NMR (500 MHz, DMSO) is δ 10.12 (s, 1H, N**H**-amide), 7.55 (d, *J* = 10.5 Hz, 1H, Ar-**H**), 7.50 (s, 1H, Ar-**H**), 7.11 (d, *J* = 10.5 Hz, 1H, Ar-**H**), 7.97 (d, *J* = 3 Hz, 2H, Ar-**H**), 6.29 (s, 1H, Ar-**H**), 3.87, 3.85 (s, 6H, -O-C**H**_3_), 3.75 (s, 6H, -O-C**H**_3_), and 2.92 (s, 3H, thiazole-C**H_3_**), and the ^13^C NMR (DMSO) is δ: 166.92 (C=O), 160.87, 160.38, 156.38, 151.76, 149.55, 146.90, 143.90, 140.83, 125.61, 125.41, 120.16, 112.50, 109.36, 98.97, 96.46, 56.18 (-O-**C**H_3_), 56.08 (-O-**C**H_3_), 55.62 (-O-**C**H_3_), and 17.65 (thiazole-**C**H**_3_**). HRMS (*m*/*z*): [M + H]+ is calculated for C_21_H_22_N_2_O_5_S as 415.1340, which is found to be 415.1334.

2-(3,4-Dimethoxyphenyl)-4-methyl-N-(4-(methylthio)phenyl)thiazole-5-carboxamide (MMH5)

For the compound 2-(3,4-dimethoxyphenyl)-4-methyl-N-(4-(methylthio)phenyl)thiazole-5-carboxamide (MMH5), its ^1^H NMR (500 MHz, DMSO) is δ 10.19 (s, 1H, N**H**-amide), 7.66 (d, *J* = 9 Hz, 2H, Ar-**H**), 7.55 (d, *J* = 10.5 Hz, 1H, Ar-**H**), 7.50 (s, 1H, Ar-**H**), 7.28 (d, *J* = 8.5 Hz, 2H, Ar-**H**), 7.11 (d, *J* = 10.5 Hz, 1H, Ar-**H**), 3.87 (s, 3H, -O-C**H_3_**), 3.84 (s, 3H, -O-C**H_3_**), 2.64 (s, 3H, thiazole-C**H_3_**), and 2.47 (s, 3H, -S-C**H**_3_), and the ^13^C NMR (DMSO) is δ166.89 (C=O), 160.24, 156.25, 151.74, 149.54, 136.55, 133.26, 127.62, 127.30, 125.62, 121.47, 120.15, 112.48, 109.34, 56.18 (-O-**C**H_3_), 56.08 (-O-**C**H_3_), 17.64 (thiazole-**C**H**_3_**), and 15.85 (-S-**C**H_3_). HRMS (*m*/*z*): [M + H]+ is calculated for C_20_H_20_N_2_O_3_S_2_ as 401.1010, which is found to be 401.1008.

### 2.4. HEK293T Cell Patch Clamp Recordings

The QIAGEN Plasmid Mini Kit was used for DNA preparation to extract high-copy plasmid DNA (up to 20 µg). The GluA2 subunit, specifically, the flip isoform obtained from S. F. Heinemann at the Salk Institute, was sub-cloned into the pRK vector for expression in Human Embryonic Kidney cells 293 (HEK293T) obtained from Sigma, Germany. HEK293T cells were chosen for their ease of cultivation and transfection efficiency. Transfection involved GluA2 and GFP-expressing constructs, and we used either jetPRIME or Lipofectamine 2000 as transfection reagents. The HEK293T cells were cultured in DMEM with 10% FBS, streptomycin, and sodium pyruvate. The cells were incubated at 37 °C with 5% CO_2_ and subcultured regularly. After 36–48 h post-transfection, highly fluorescent cells were selected for recordings, as explained in our previous work [31].

Electrophysiological recordings were carried out using a patch clamp setup equipped with a rapid perfusion system. Recordings were performed at 22 °C with a membrane potential of −60 mV, amplified using an integrated patch amplifier, and digitized. Patch pipettes had a resistance range of 2–4 MΩ and were filled with a specific solution. The extracellular medium composition was standardized. A sample size of six viable cells was used to calculate the mean inhibition. The solution exchange time was approximately 500 milliseconds; this has been detailed further in previous works [32,33].

Data were analyzed using Igor Pro7 software. Receptor desensitization and deactivation rates were determined by fitting the current decay using a double exponential model. Weighted mean time constants (τ_w_) were calculated. Statistical analyses were performed using ANOVA to assess differences among chemical compounds, with significance levels indicated.

### 2.5. Molecular Docking Studies

In this research, an integrated single-click docking method was employed to perform molecular docking of the most active compound (**MMH5**). The compound’s chemical structure was sketched and then docked with the **7RZ5** protein structure (PDB code: 7Rz5), which was downloaded from the RCSB repository server. Subsequently, the protein structures were analyzed with PyMOL v2.5. The native ligand, non-protein atoms, and crystallographic waters were removed. Polar hydrogen atoms were added to the previous structure, and sidechains were protonated, assuming a physiological pH of 7.4 using an H++ server. Using the 1-click docking free server (https://mcule.com/apps/1-click-docking accessed on 18 October 2023), the binding site as well as the binding site dimensions were identified according to the literature. Additionally, the interaction profile was analyzed between the compound MMH5 and the receptor using the Protein–Ligand Interaction Profiler (PLIP) service [34,35].

### 2.6. Cell Culture and Cytotoxicity Assay (MTS)

All of the thiazole derivatives’ cytotoxicity was investigated using cultured cell lines, including HepG2, MCF-7, HeLa, CaCo-2, cancer cell lines, and the normal hepatic cell line LX-2, which were grown separately in RPMI-1640 media (Sigma, Norwich, UK). The media were supplemented with 1% L-glutamine (Sigma, London, UK), 1% penicillin/streptomycin antibiotics (BI, New Delhi, India), and 10% fetal bovine serum. These cells were cultured under humid conditions with 5% CO_2_ at 37 °C. In 96-well plates, cells were seeded at a density of 2.6 × 10^4^ cells per well. After 48 h, cancer cells were exposed to thiazole derivatives (MMH1-5) at one concentration (50 µM) for 24 h. Cell viability was assessed using the CellTiter 96^®^ Aqueous One Solution Cell Proliferation (MTS) Assay from Promega Corporation, Madison, WI, USA, following the manufacturer’s instructions. Following the treatment, 20 µL of MTS solution was added to each well containing 100 µL of medium, and the plates with thiazole derivatives were incubated at 37 °C for 2 h. Absorbance was measured at 490 nm [36,37].

## 3. Results and Discussion

### 3.1. Chemistry

Following the procedure outlined in Scheme 1, a sequence of thiazole derivatives, namely **MMH1-5**, were synthesized. The synthesis initiation involved dissolving 2-(3,4-dimethoxyphenyl)-4-methylthiazole-5-carboxylic acid in dichloromethane (DCM). Subsequently, a blend of dimethylaminopyridine (DMAP) and EDCI was introduced, and the components were thoroughly mixed within an inert gas environment. The corresponding aniline derivative was added after a 30 min incubation period in each experiment. The resulting reaction was allowed to stir for 48 h. Following this reaction, an extraction process was conducted by utilizing a 32% hydrochloric acid (HCl) solution, followed by treatment with anhydrous sodium sulfate. The HRMS values for all the synthesized compounds confirmed their respective molecular weights. The ^1^H-NMR spectra verified the synthesis of these compounds, with each compound displaying a single peak for the N-H amide proton around 10.05 ppm. A signal peak representing nine protons was also evident for the compound featuring a t-butyl substituent at 1.29 ppm. The ^13^C-NMR spectrum exhibited a distinctive signal for the carbonyl group at around 166 ppm, for methoxy groups at around 56, and a signal was observed for all compounds at 17 ppm, which represents the methyl group of the thiazole ring substituted methyl.

### 3.2. Thiazole Derivatives’ Effects on GluA2 Currents and Kinetics: Unveiling Desensitization and Deactivation Dynamics

**Figure 2:**
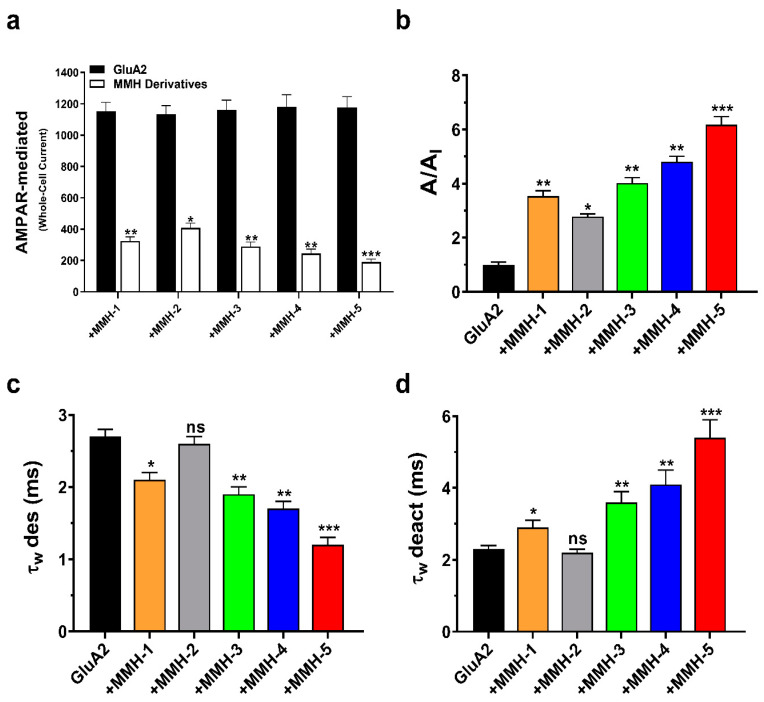
Modulation of GluA2 subunit-driven whole-cell current and kinetics by MMH compounds. (**a**) Whole-cell current amplitude (in picoamperes) illustrating the impact of glutamate alone (black) on the GluA2 subunit and the effect of five MMH compounds (at a concentration of 16 µM) (white). (**b**) Calculated *A/A_I_* ratio, where A represents the current with glutamate alone, and A_I_ is the inhibitory current observed after applying the MMH compounds. (**c**) Effect of the compounds on the desensitization rate (τ_w_ des). MMH-1 (orange), MMH-2 (gray), MMH-3 (green), MMH-4 (blue), and MMH-5 (red) are shown. (**d**) Effect of the compounds on the deactivation rate (τ_w_ deact). MMH-1 (orange), MMH-2 (gray), MMH-3 (green), MMH-4 (blue), and MMH-5 (red) are displayed. The experiments were conducted under controlled conditions, including a pH of 7.4, a temperature of 22 °C, and a voltage of −60 mV. Data are presented as the mean ± standard error of the mean (SEM), with *n* = 6 representing the number of patch cells tested. Statistical significance of one-way ANOVA results: (*) *p* < 0.05, (**) *p* < 0.01, (***) *p* < 0.001; ns, not significant.

The effects of thiazole carboxamide derivatives were investigated on AMPA-R homomeric subunit GluA2 whole-cell currents, as well as their impact on deactivation (τ_w_ deact) and desensitization rates (τ_w_ des). The detailed results are presented in Tables S1–S5. Figure 2a illustrates the alterations in GluA2 whole-cell currents, following exposure to the thiazole derivatives. The A/AI ratio was calculated to quantify these changes, representing the current amplitudes before and after thiazole derivative exposure (Figure 2b). Compound **MMH-5** demonstrated the most substantial effect, causing a remarkable 6-fold decrease in current amplitude. Following closely, **MMH-4** induced a 5-fold reduction, while MMH-3 resulted in a 4-fold decrease. MMH-2 and MMH-1 exhibited comparatively milder effects, reducing GluA2 activity by nearly 3-fold.

Shifting our focus toward the kinetics of the GluA2 subunit, interesting dynamics were observed for the thiazole derivatives. Specifically, **MMH-5** and **MMH-4** led to a notable increase in the deactivation rate, approximately 3-fold (Figure 2d). In contrast, MMH-3 had a milder effect, elevating the deactivation rate by 1.5-fold. Regarding desensitization rates (Figure 2c), **MMH-5** demonstrated a significant reduction, reducing the desensitization rate approximately by half. MMH-4 and MMH-3 also exhibited a reduction in desensitization, though to a smaller degree, each resulting in approximately a 1-fold decrease. These findings highlight the distinct effects of the thiazole derivatives on both GluA2 whole-cell currents and the kinetics of the GluA2 subunit, shedding light on the potential modulatory roles of these compounds.

Compounds with heterocycles have garnered substantial attention in recent years due to their potential therapeutic applications. These interactions can lead to anticonvulsant properties, with carbonyl groups, heteroatoms, and aromatic or heterocyclic rings playing pivotal roles in these pharmacophore groups. In our previous research on 2-oxo-3*H*-benzoxazole derivatives, these compounds exhibited essential pharmacophores that interact with amino acids in the binding site of AMPA receptor subunits, resulting in receptor inhibition and potential anticonvulsant effects [38]. Previous research has identified critical residues surrounding the binding pocket, including the pre-M1, M3, and M4 helices, as well as the S2-M4 linker. These residues are expected to play a crucial role in compound binding, as evidenced by interactions with known antagonists like DNQX and ZK200775. Our analysis suggests that these residues are also pivotal in interacting with our thiazole derivatives, which exhibit AMPA receptor antagonistic effects akin to DNQX and ZK200775. This suggests that thiazole compounds may function as AMPA receptor antagonists, likely employing a binding mechanism similar to the aforementioned antagonists [39,40].

The AMPA receptor-affecting compounds encompass the primary pharmacophore groups, including carbonyl, hetero atoms, and halogenated aromatic or heterocyclic rings. These groups are capable of facilitating various binding interactions with AMPA receptor amino acids, such as hydrogen, hydrophobic, and π–π interactions involving amino acids like ASN791, PHE623, PRO520, and LEU620 [41,42]. The carbonyl functional group and/or hetero atoms, like oxygen, can establish hydrogen bonds, while aromatic or heterocyclic rings contribute to hydrophobic interactions [41]. These pharmacophores are fundamental for substantial binding interactions with various AMPA receptor subtypes. Each compound in our study incorporates a thiazole moiety, facilitating π–π or hydrogen bonding interactions, along with a carbonyl amide group capable of establishing hydrogen bonding interactions.

The investigation into compounds that can modulate the behavior of GluA2-containing AMPA receptors is of paramount importance, given the physiological and pathological implications of these receptors. The study at hand delves into a novel class of compounds known as thiazole carboxamide derivatives. These compounds exhibit structural similarities to well-established pharmacologically active agents, featuring the shared thiazole ring like Riluzole, renowned for its neuroprotective properties in conditions such as amyotrophic lateral sclerosis [22,23]. Similar to our compounds, the thiazolamide derivatives were synthesized to explore their potential as mGluR1 antagonists. Notably, they exhibited substantial activity against mGluR1—although it faced challenges related to metabolic stability and water solubility [24]—as well as thiazole-4-carboxamide derivatives, as mGluR5a receptor antagonists were displaying a favorable mGluR5a binding affinity [25]. Previous research from our team involved the synthesis of heterocyclic-based curcumin compounds featuring methoxy substitutions on the phenyl ring, with notable activity on GluA2 receptor subtypes [11].

### 3.3. Molecular Docking and SAR

**Figure 3:**
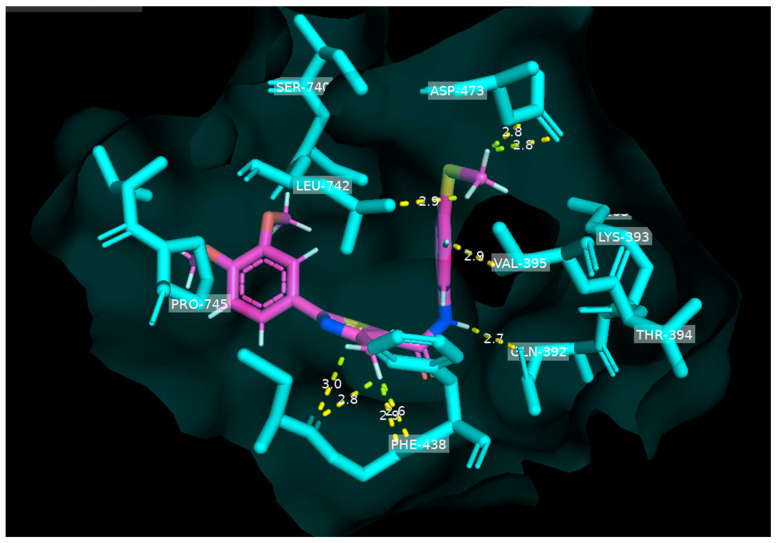
Computational molecular docking of **MMH-5** compound within the binding site of GluA2-AMPA receptor (PDB ID: 7RZ5): analyzing hydrogen bonds, bond distances, and binding site surface interactions.

The compound with the greatest efficacy, MMH-5, was chosen for molecular docking studies to predict its potential binding interactions with the AMPA receptor. Based on the findings from Figure 3 and Table 1, it was observed that approximately nine binding interactions occurred between MMH-5 and specific amino acids, including GLN-293, ASP-473, PHE-438, VAL-395, and LEU-742. These interactions were characterized by ideal distances, all within 3.0 Å or less. Notably, a robust hydrogen bond was formed between GLN-293 and the H-amide group at a distance of just 2.7 Å. Detailed information on the other binding interactions and their respective distances can be found in Table 1 and Figure 3.

It is noteworthy that the fundamental pharmacophore groups previously discussed concerning compounds affecting the AMPA receptor and SAR studies, such as carbonyl, hetero atoms, and heterocyclic rings, play a pivotal role in facilitating a diverse spectrum of binding interactions with AMPA receptor amino acids. In the case of the compound **MMH5**, all of these crucial features were present. The amide group formed a hydrogen bond with GLN-392; the heterocyclic (thiazole) engaged in four binding interactions with PHE-438; the phenyl-amide ring presented a more important binding interaction than the second phenyl ring; and the presence of a methylthio group was optimal for establishing three binding interactions with ASP-473 and LEU-742 amino acids. It became evident that the inclusion of larger substituents such as trimethoxy groups or tert-butyl, as seen in **MMH1** and **MMH2**, respectively, did not effectively facilitate binding interactions. In fact, they introduced a restrictive effect that resulted in diminished activity with GluA2 amino acids and the absence of three binding interactions. On the contrary, the presence of smaller substituents like methylthio proved to be highly effective in promoting binding interactions with ASP-473 and LEU-742, leading to robust binding interactions and the potent activity of compound **MMH5**.

The intricacies of thiazole compounds’ effects on AMPA receptors featuring GluA2 subunits provide an intriguing focus for neuroscience and pharmacology. Our data elucidate a compelling correlation between the molecular architecture of thiazole derivatives and their consequential modulation of receptor kinetics. Specifically, **MMH1**, **MMH3**, and **MMH4,** each with their unique configurations of 3,4-dimethoxyphenyl and additional substituents like 3,4,5-trimethoxyphenyl, demonstrated substantial decreases in amplitude along with significant changes in deactivation and desensitization times (t deact and t des) when compared to glutamate alone. These structural nuances, particularly the presence and positioning of methoxy groups on the phenyl rings, may enhance the electron-donating abilities of these molecules. This likely influences their interaction affinity and kinetics with the GluA2 subunits, echoing the behaviors of known AMPA receptor modulators like talampanel and GYKI 53655. The unique kinetic behavior of **MMH5,** characterized by a 4-(methylthio)phenyl moiety, underscores the impact of even slight structural alterations on receptor modulation. It was clear that these thiazole carboxamide derivatives have structural similarities to established pharmacologically active agents. This unique approach presents the opportunity to explore these compounds as potential modulators of GluA2-containing AMPA receptors, a focus that aligns with the growing understanding of the physiological and pathological significance of these receptors. The study’s emphasis on the pharmacophore groups shared by these compounds and their potential interactions with AMPA receptor amino acids adds an innovative layer to the research. Furthermore, the data delve into the critical residues involved in compound binding, shedding light on the compounds’ possible mechanisms of action. The findings extend to the modulation of receptor kinetics, highlighting how subtle structural alterations can significantly impact receptor behavior. This holistic approach not only enhances our understanding of AMPA receptor modulation, but also opens doors to the development of novel therapeutics in the fields of neurobiology and drug discovery.

### 3.4. Cytotoxicity Results

**Figure 4:**
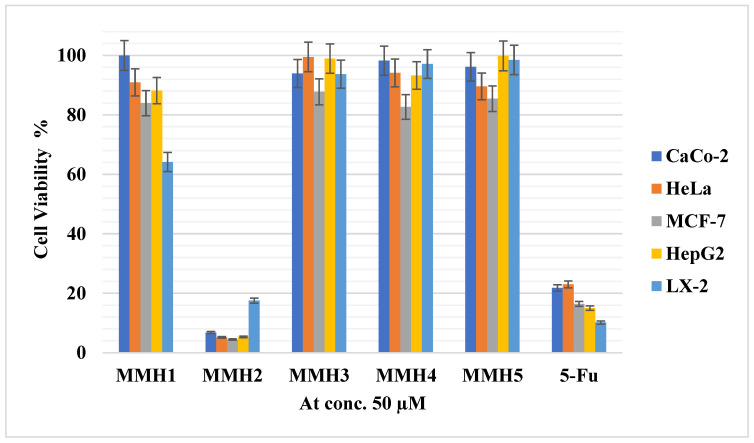
Cell viability values at 50 µM on various cell lines of the thiazole derivatives in comparison with positive control.

Cytotoxicity assessments were conducted on the thiazole derivatives to ensure their safe use at a concentration approximating the effective dose of 15 µM on the GluA2 receptor. Figure 4 presents the cell viability percentages at a 50 µM concentration for various cancer cell lines and a normal cell line (LX-2). Notably, compound **MMH2** (N-(4-(tert-butyl)phenyl)-2-(3,4-dimethoxyphenyl)-4-methylthiazole-5-carboxamide) displayed remarkable cytotoxicity, with cell viability values falling below 6.79% for all cancer cell lines and at 17.52% for the normal cell line (LX-2) at 50 µM concentration, in comparison with positive-control anticancer drug 5-fluorouracil cell viability values falling below 22.99% for all cancer cell lines and at 10.17% for the normal cell line (LX-2) at the same concentration. In contrast, the cell viability percentages for the other compounds exceeded 80% for nearly all compounds across a wide range of cell lines. These values were compared to the positive control, the anticancer drug 5-fluorouracil. It is particularly noteworthy to highlight the cytotoxicity of **MMH5**, as it stood out as the most potent compound targeting the GluA2 receptor subtype, with cell viability percentages surpassing 85.44% across all cancer and normal cell lines, affirming its safety for use as a neuroprotective agent on the AMPA receptor. The intriguing anticancer activities observed for compound **MMH2** against all cancer cell lines can be attributed to the presence of the tert-butyl substituent on the phenyl ring. This structural feature enhances the compound’s lipophilicity, facilitating its easy cell membrane penetration and interaction with intracellular targets.

## 4. Conclusions

In summary, our research investigates the impact of thiazole carboxamide derivatives on GluA2 AMPA receptors. These experiments have provided valuable insights into the complex interplay between these chemicals and receptor kinetics, enhancing our understanding of their potential as regulators of AMPA receptor activity. **MMH5** is notable for its strong negative allosteric modulation effects, which substantially impact both the amplitude and kinetics of the receptor. This underscores the potential therapeutic relevance of **MMH5**. Furthermore, our extensive evaluations of cytotoxicity have provided evidence supporting the safety of these compounds at concentrations proximate to the efficacious dosage of the GluA2 receptor. However, it is worth noting that compound **MMH2** exhibits substantial cytotoxic properties on cancer cell lines, thereby demonstrating its potential as an anticancer agent. This effect is attributed to the distinctive structural characteristic of a tert-butyl substituent on the phenyl ring, which enhances its ability to penetrate cellular membranes and interact with intracellular targets. As mentioned earlier, the results provide a robust basis for future investigations and research initiatives to develop customized therapeutics for neurological disorders in which GluA2 signaling plays a crucial role. The observed effects of these thiazole derivatives and the encouraging cytotoxicity outcomes provide intriguing opportunities for enhancing tailored therapies and therapeutic approaches in neurology and pharmacology. The significant regulatory impacts of **MMH5** highlight the possibility of focused interventions in many neurological illnesses, signaling the advent of a new era in tailored pharmacology. The conclusion of this research underscores the potential for future work in the fields of neurology and pharmacology. This includes in-depth investigations into the mechanistic details of thiazole carboxamide derivatives’ interactions with GluA2 AMPA receptors and the synthesis of novel compounds for enhanced pharmacological effects. Moreover, the transition to preclinical studies and clinical trials is essential for evaluating the compounds’ safety and efficacy as potential therapeutic agents. Additionally, the broad applications of these compounds in treating neurological disorders and their potential for customized pharmacological interventions mark a promising path for future research and therapeutic development.

## Figures and Tables

**Table 1:** Detailed interactions and binding scores of MMH-5 compound within the binding pocket of GluA2-AMPA (PDB ID: 7RZ5) target protein.

Hydrogen Bonds	Bond Distance (Å)	Hydrophobic Bonds	Bond Distance (Å)	Docking Score
GLN-392 ASP-473 PHE-438	2.7 2.8 2.8–3.0	VAL-395 PHE-438 LEU-742 ASP-473	2.9 2.6–2.9 2.9 2.8	−7.3

## Data Availability

This published article and its Supplementary Materials include all data generated or analyzed during this study.

## Supplementary Materials

The following supporting information can be downloaded at: https://www.mdpi.com/article/10.3390/biom13121694/s1, Figure S1: NMR Spectrum of MMH1; Figure S2: NMR Spectrum of MMH2; Figure S3: NMR Spectrum of MMH3; Figure S4: NMR Spectrum of MMH4; Figure S5: NMR Spectrum of MMH5; Table S1: Whole-cell recordings for compound MMH1; Table S2: Whole-cell recordings for compound MMH2; Table S3: Whole-cell recordings for compound MMH3; Table S4: Whole-cell recordings for compound MMH4; Table S5: Whole-cell recordings for compound MMH5.
