# Supplementary material for: Thiazole Derivatives as Modulators of GluA2 AMPA Receptors: Potent Allosteric Effects and Neuroprotective Potential
Source: Biomolecules. 2023 Nov 23;13(12):1694. doi: 10.3390/biom13121694 (PMC10741633; doi:10.3390/biom13121694)

Supplementary Material

Thiazole Derivatives as Modulators of GluA2 AMPA Receptors: Potent Allosteric Effects and Neuroprotective Potential

Mohammed Hawash

Department of Pharmacy, Faculty of Medicine and Health Sciences, An-Najah National University, Nablus, Palestine.

*Corresponding author: **Mohammed Hawash**, Department of Pharmacy, Faculty of Medicine and Health Sciences, An-Najah National University, Nablus, PALESTINE, orcid.org/0000-0001-5640-9700; Phone: +972569939939; Email: mohawash@najah.edu.

Figures and Tables **Pages**

Supplemental Table S1 Whole-cell recordings for compound MMH-1.………… 3

Supplemental Table S2 Whole-Cell Recordings for Compound MMH-2……..… 3

Supplemental Table S3 Whole-Cell Recordings for Compound MMH-3……….. 3

Supplemental Table S4 Whole-Cell Recordings for Compound MMH-4……….. 4

Supplemental Table S5 Whole-Cell Recordings for Compound MMH-5………… 4

Figure S1. NMR Spectrum of MMH1 ……….……………………………………. 5

Figure S2. NMR Spectrum of MMH2……………………..…………………….. 6

Figure S3. NMR Spectrum of MMH3…..………………………………………….. 7

Figure S4. NMR Spectrum of MMH4……………………………………..…….. 8

Figure S5. NMR Spectrum of MMH5……………………………………..…….. 9

Supplemental Table S1 Whole-Cell Recordings for Compound MMH-1

Receptor Name/Compounds abbreviation	GluA2 (Glutamate Alone)	MMH-1	Applying Glutamate Alone After MMH-1	n	A/A_I
Amplitude (pA)	1151±59	325±26**	1154±60	6	3.54±0.2
t deact (ms)	N/R	2.9±0.2*	N/R	6	N/R
t des (ms)	N/R	2.1±0.1*	N/R	6	N/R

Supplemental Table S2 Whole-Cell Recordings for Compound MMH-2

Receptor Name/Compounds abbreviation	GluA2 (Glutamate Alone)	MMH-2	Applying Glutamate Alone After MMH-2	n	A/A_I
Amplitude (pA)	1135±53	408±32*	1139±56	6	2.78±0.1
t deact (ms)	N/R	2.2±0.1^{ns}	N/R	6	N/R
t des (ms)	N/R	2.6±0.1^{ns}	N/R	6	N/R

Supplemental Table S3 Whole-Cell Recordings for Compound MMH-3

Receptor Name/Compounds abbreviation	GluA2 (Glutamate Alone)	MMH-3	Applying Glutamate Alone After MMH-3	n	A/A_I
Amplitude (pA)	1161±62	289±29**	1163±64	6	4.02±0.2
t deact (ms)	N/R	3.6±0.3**	N/R	6	N/R
t des (ms)	N/R	1.9±0.1**	N/R	6	N/R

Supplemental Table S4 Whole-Cell Recordings for Compound MMH-4

Receptor Name/Compounds abbreviation	GluA2 (Glutamate Alone)	MMH-4	Applying Glutamate Alone After MMH-4	n	A/A_I
Amplitude (pA)	1182±76	246±27**	1178±75	6	4.81±0.2
t deact (ms)	N/R	4.1±0.4**	N/R	6	N/R
t des (ms)	N/R	1.7±0.1**	N/R	6	N/R

Supplemental Table S5 Whole-Cell Recordings for Compound MMH-5

Receptor Name/Compounds abbreviation	GluA2 (Glutamate Alone)	MMH-5	Applying Glutamate Alone After MMH-5	n	A/A_I
Amplitude (pA)	1175±71	190±20***	1174±66	6	6.18±0.3
t deact (ms)	2.3±0.1	5.4±0.5***	N/R	6	N/R
t des (ms)	2.7±0.1	1.2±0.1***	N/R	6	N/R

FigureS1. 2-(3,4-dimethoxyphenyl)-4-methyl-N-(3,4,5-trimethoxyphenyl)thiazole-5-carboxamide (MMH1)

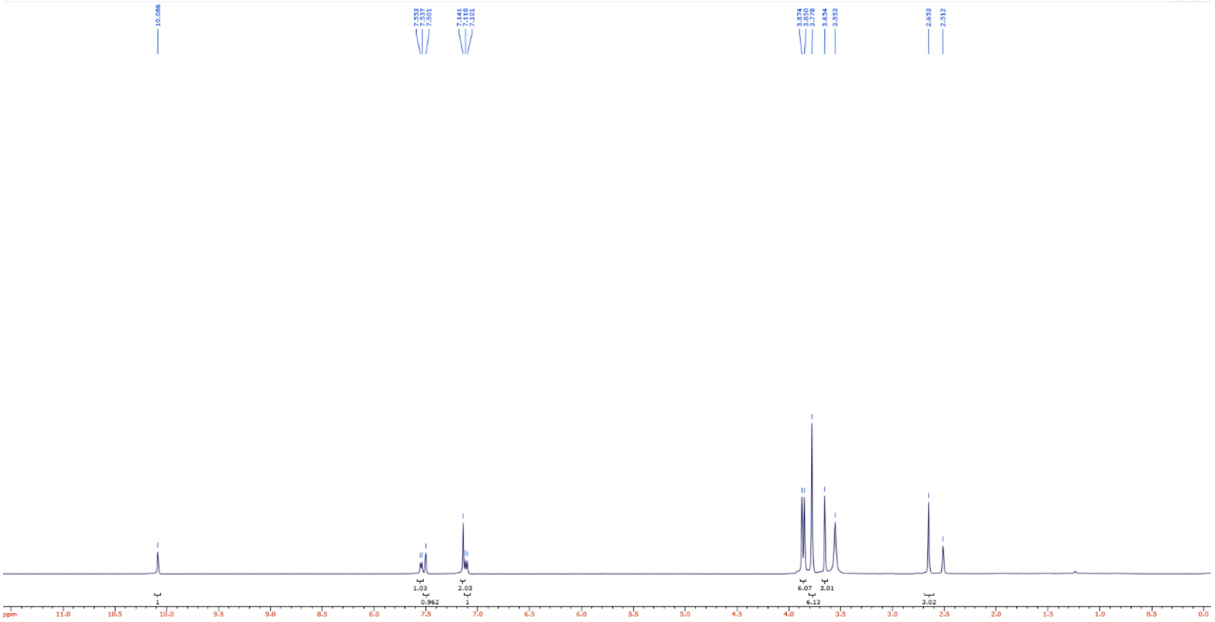

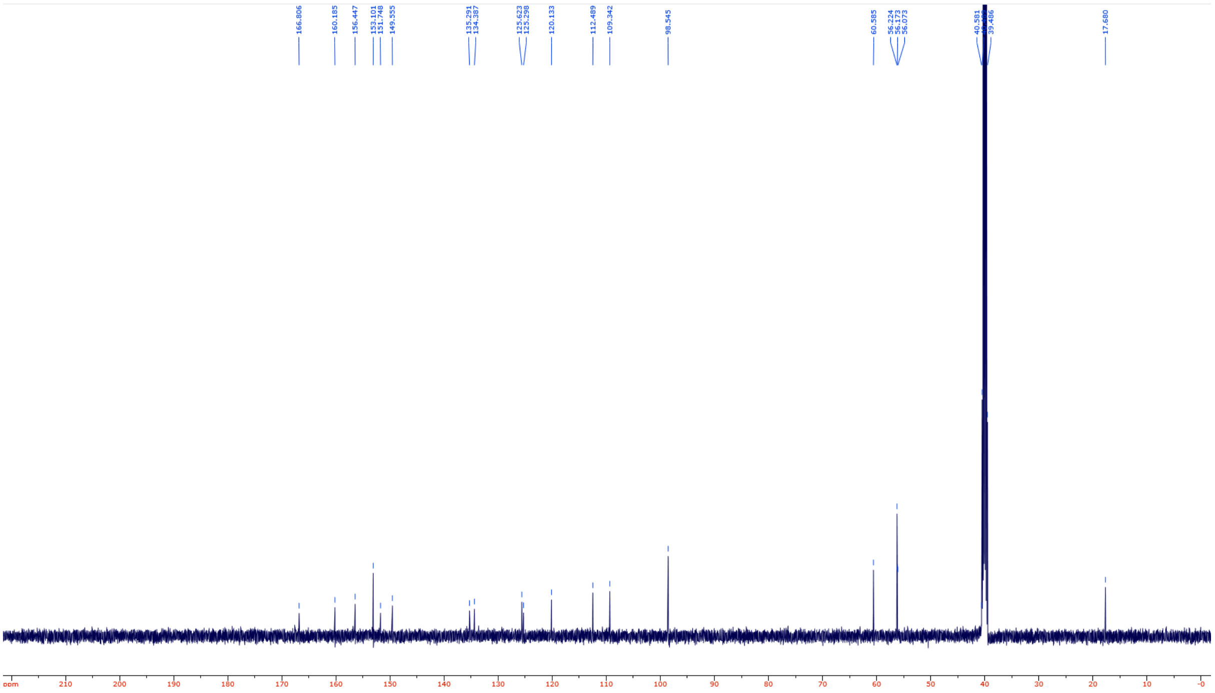

Figure S2. N-(4-(tert-butyl)phenyl)-2-(3,4-dimethoxyphenyl)-4-methylthiazole-5-carboxamide (MMH-2)

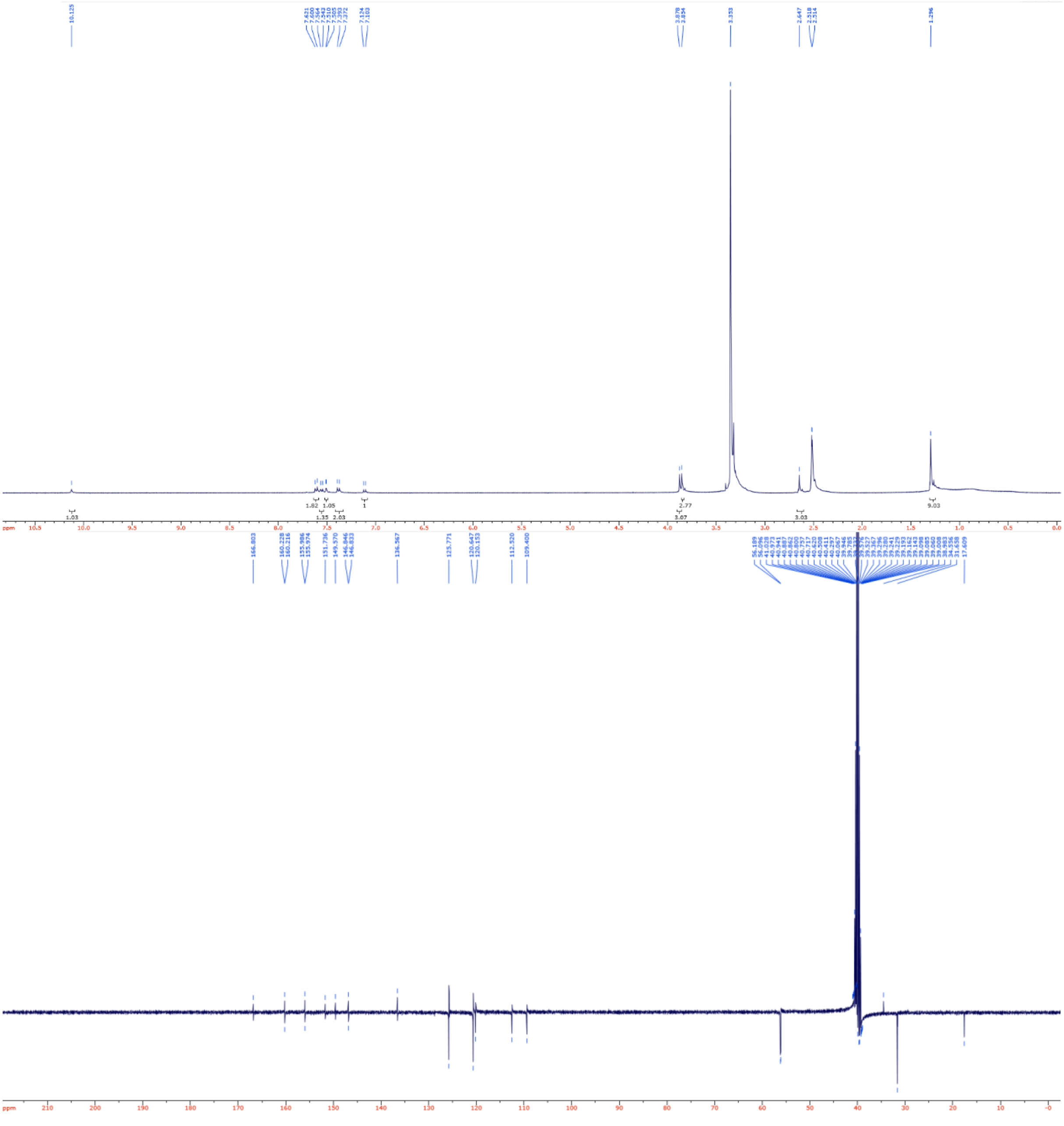

Figure S3. N,2-bis(3,4-dimethoxyphenyl)-4-methylthiazole-5-carboxamide (MMH3)

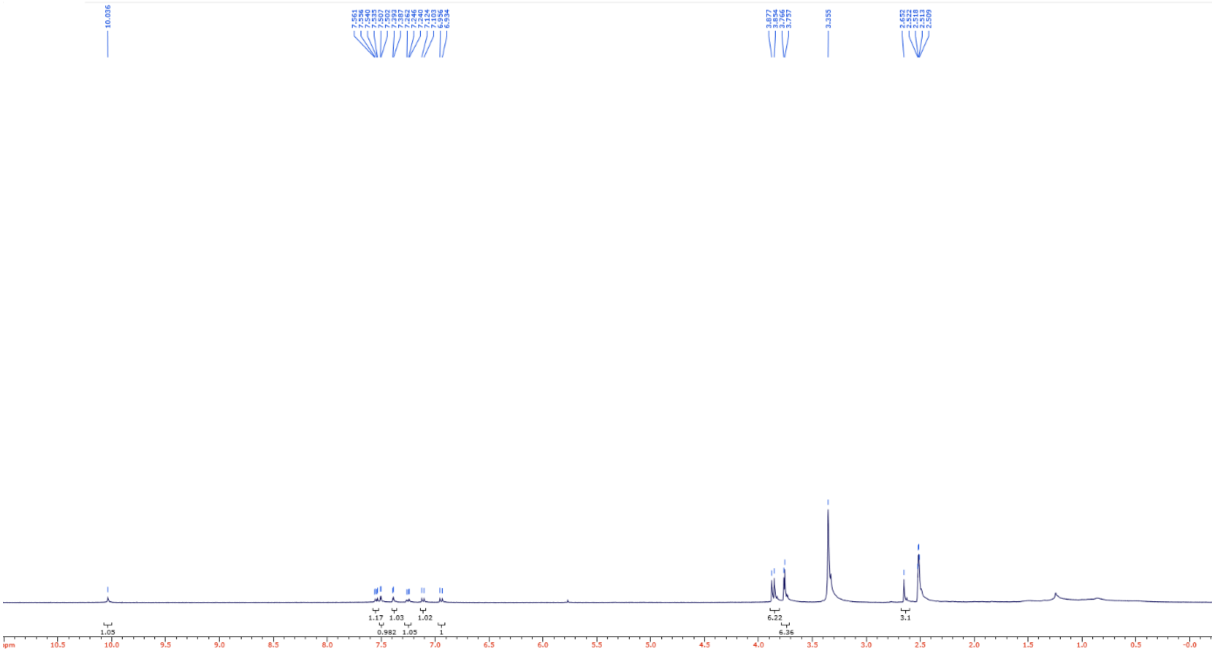

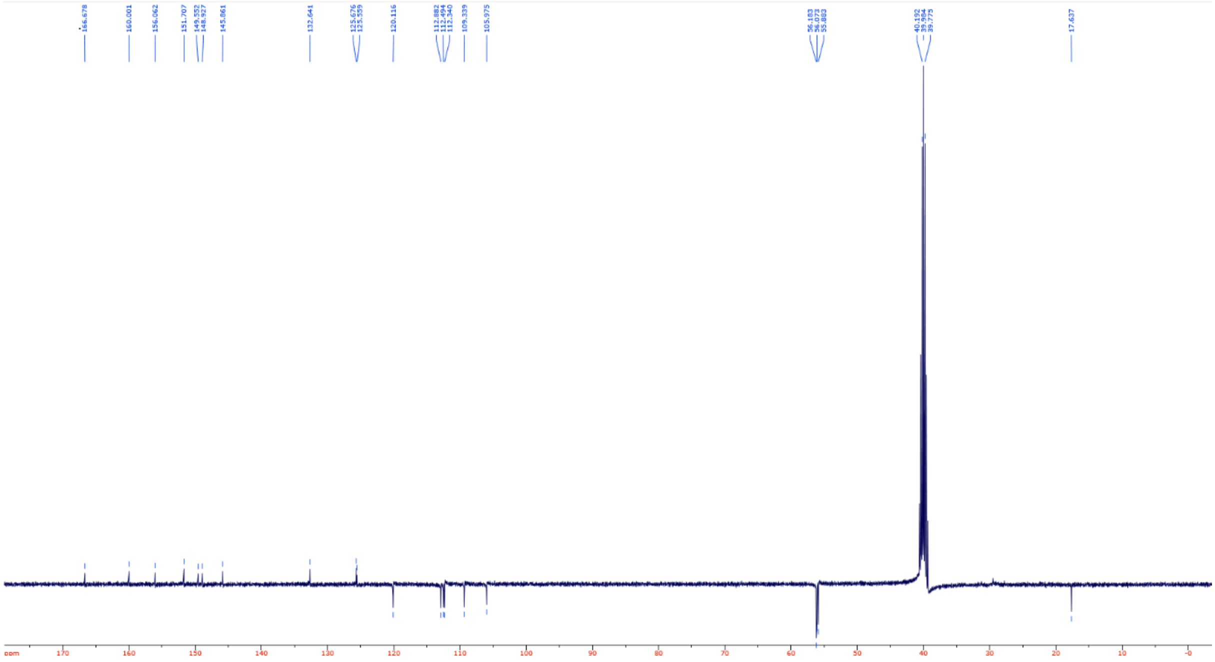

Figure S4. **2-(3,4-dimethoxyphenyl)-N-(3,5-dimethoxyphenyl)-4-methylthiazole-5-carboxamide (MMH4)**

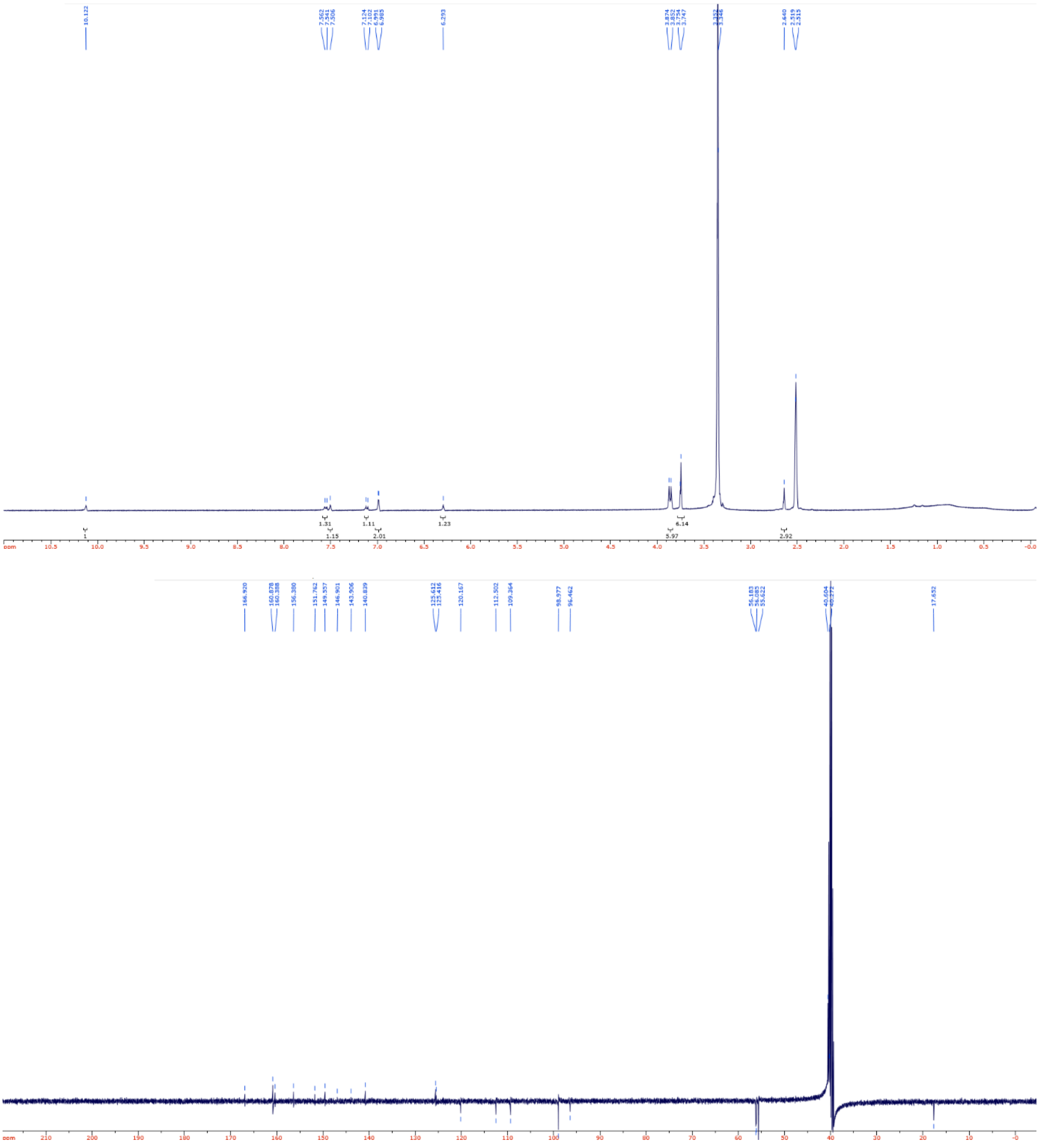

Figure S5. 2-(3,4-dimethoxyphenyl)-4-methyl-N-(4-(methylthio)phenyl)thiazole-5-carboxamide (MMH5)

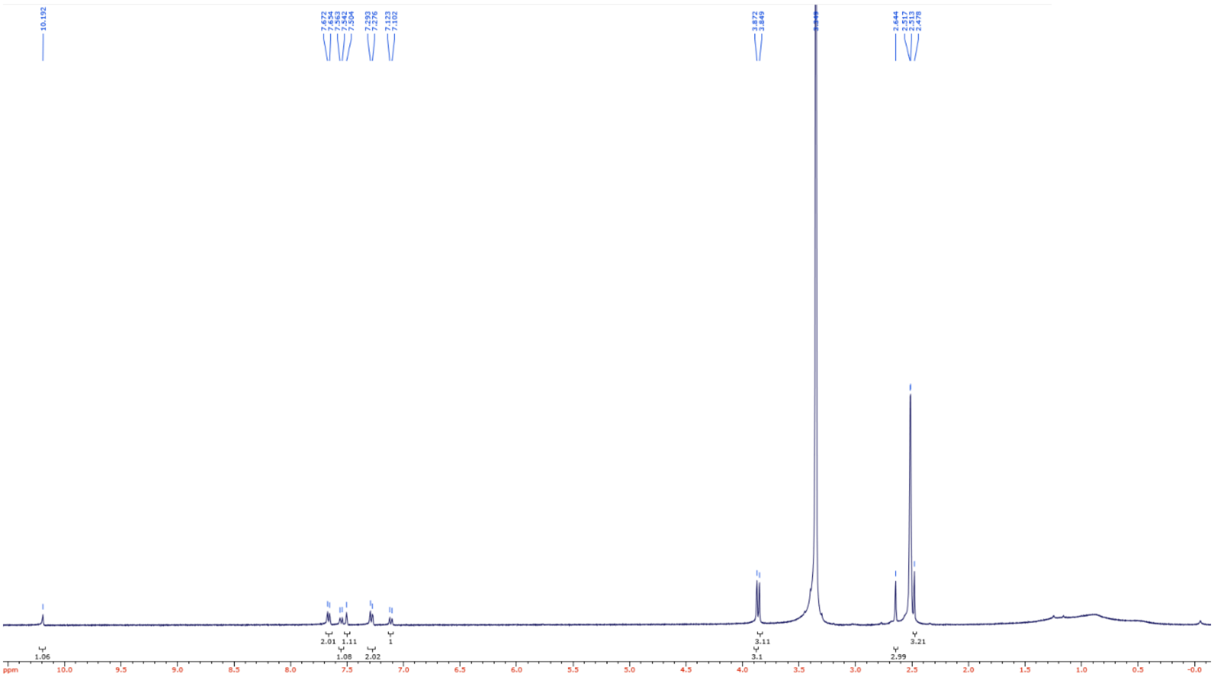

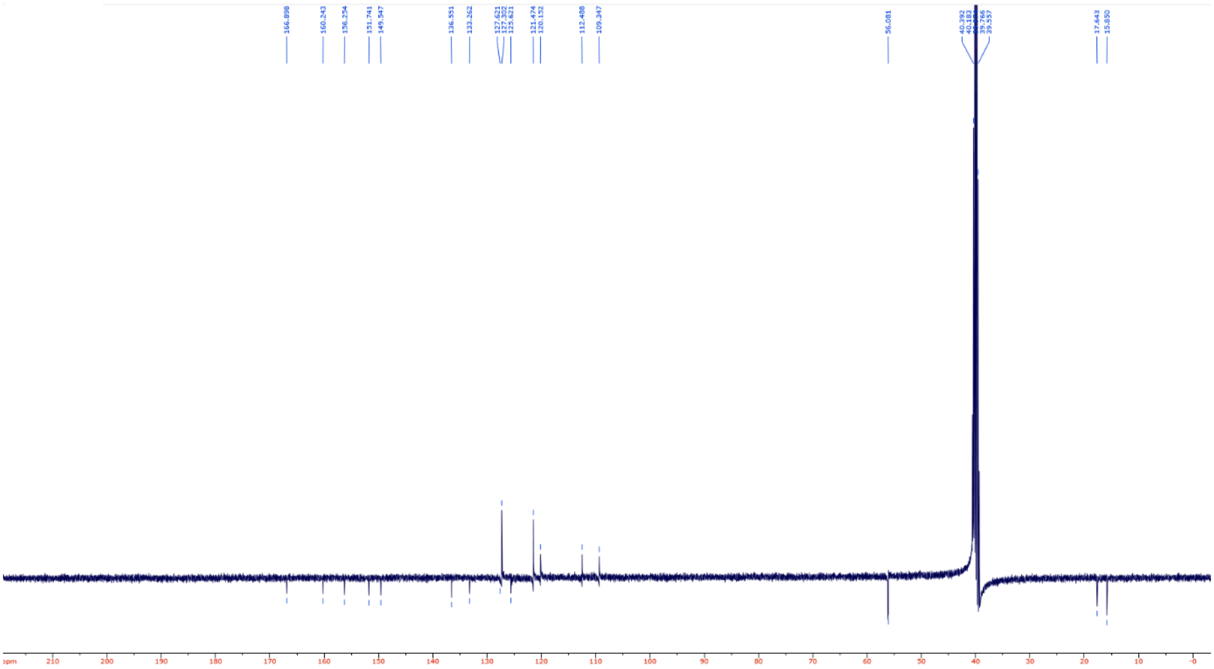

Supplement: Supplementary file 1 [file biomolecules-13-01694-s001.zip › biomolecules-2699347-supplementary.pdf]
